# 916. A Retrospective Review of Suicide by Self-immolation: Comparing Pre-, During, and Post-COVID-19 Data

**DOI:** 10.1093/jbcr/irag033.441

**Published:** 2026-04-06

**Authors:** Brett Opelt, Shawn Tejiram, Jeffrey W Shupp, Taryn E Travis, Jamie G Oh, Daniel M Schneider

**Affiliations:** The Burn Center at MedStar Washington Hospital Center, Bowie, MD; MedStar Washington Hospital Center, Washington, DC; MedStar Washington Hospital Center, Washington, DC; The Burn Center at MedStar Washington Hospital Center, Bowie, MD; MedStar Washington Hospital Center, Washington, DC; The Burn Center at MedStar Washington Hospital Center, Bowie, MD

## Abstract

**Introduction:**

The COVID-19 pandemic had a significant impact on mental health. With the increased incidence of mental health concerns, there has been an increase in the trends of suicide attempts and deaths globally. A small percentage of suicide cases included the rare method of self-harm and self-immolation. Previous research examining the incidence rates of self-immolation as a form of suicide during and after the COVID-19 pandemic is limited in the U.S. The objective of this study was to compare pre-, during, and post-COVID-19 pandemic rates of suicide through self-immolation by reviewing information entered and collected into a single institution’s burn registry data.

**Methods:**

A retrospective review of burn registry data at a single ABA-verified burn center focused on data identified for patient admissions with self-immolation over ten years, which included the period of the pandemic (2014-2024). Three groups were created: pre-, during, and post-pandemic. ANOVA was used to compare the means between demographics, mortality, etiology, and increases of incidence in self-immolation.

**Results:**

Of the total of 87 cases, significantly higher rates of mortality by self-immolation found after the pandemic. As well, the use of gasoline etiology subcategory was significantly higher during and after the pandemic. There was no statistically significant difference in self-immolation rates between all three periods.

**Conclusions:**

The retrospective review at a single ABA-verified burn center did not see a significant increase in incidence rates for suicide by self-immolation. Changes in mechanism were seen during and after the pandemic, with the use of gasoline and higher mortality in self-immolation.

**Applicability of Research to Practice:**

Understanding incidence rates, especially during an event as significant as the pandemic, would allow burn centers and other mental health organizations to develop programming addressing self-immolation as a mechanism of self-harm or suicide.

**Funding for the study:**

N/A.

